# D-sORF: Accurate Ab Initio Classification of Experimentally Detected Small Open Reading Frames (sORFs) Associated with Translational Machinery

**DOI:** 10.3390/biology13080563

**Published:** 2024-07-26

**Authors:** Nikos Perdikopanis, Antonis Giannakakis, Ioannis Kavakiotis, Artemis G. Hatzigeorgiou

**Affiliations:** 1 Department of Electrical and Computer Engineering, University of Thessaly, 38221 Volos, Greece; 2 Department of Informatics and Telecommunications, National and Kapodistrian University of Athens, 15784 Athens, Greece; 3 Department of Computer Science and Biomedical Informatics, University of Thessaly, 38221 Volos, Greece; ikavakiotis@gmail.com; 4 Department of Molecular Biology and Genetics, Democritus University of Thrace, 68100 Alexandroupolis, Greece; antgian@mbg.duth.gr; 5 University Research Institute of Maternal and Child Health and Precision Medicine, National and Kapodistrian University of Athens, 11527 Athens, Greece; 6 Hellenic Pasteur Institute, 11521 Athens, Greece

**Keywords:** small open reading frames, sORF, machine learning, motif prediction, ribosome sequencing, genomic annotation

## Abstract

**Simple Summary:**

Small open reading frames (sORFs; fewer than 300 nucleotides or fewer than 100 amino acids) are short DNA sequences that can regulate cellular processes or produce functional peptides. Identifying these sORFs, especially in non-genic regions, remains challenging despite advances in sequencing technology. To address this, we developed D-sORF, a machine-learning framework that predicts coding sORFs using the nucleotide context and motifs around start codons. D-sORF achieves 94.74% precision and 92.37% accuracy, outperforming experimental methods such as ribosome sequencing (Ribo-Seq) in identifying peptide-producing transcripts and filtering out false positives. Unlike traditional conservation-based methods, D-sORF’s significant advantage is recognizing sORFs with low sequence similarity. Its robust prediction capabilities make it a valuable tool for researchers. It can enhance our understanding of sORFs’ roles, potentially leading to discoveries in terms of gene regulation and new therapeutic targets. By accurately distinguishing small coding sequences from non-coding ones, D-sORF significantly contributes to genomic research and its applications in medicine and biotechnology.

**Abstract:**

Small open reading frames (sORFs; <300 nucleotides or <100 amino acids) are widespread across all genomes, and an increasing variety of them appear to be translating from non-genic regions. Over the past few decades, peptides produced from sORFs have been identified as functional in various organisms, from bacteria to humans. Despite recent advances in next-generation sequencing and proteomics, accurate annotation and classification of sORFs remain a rate-limiting step toward reliable and high-throughput detection of small proteins from non-genic regions. Additionally, the cost of computational methods utilizing machine learning is lower than that of biological experiments, and they can be employed to detect sORFs, laying the groundwork for biological experiments. We present D-sORF, a machine-learning framework that integrates the statistical nucleotide context and motif information around the start codon to predict coding sORFs. D-sORF scores directly for coding identity and requires only the underlying genomic sequence, without incorporating parameters such as the conservation, which, in the case of sORFs, may increase the dispersion of scores within the significantly less conserved non-genic regions. D-sORF achieves 94.74% precision and 92.37% accuracy for small ORFs (using the 99 nt medium length window). When D-sORF is applied to sORFs associated with ribosomes, the identification of transcripts producing peptides (annotated by the Ensembl IDs) is similar to or superior to experimental methodologies based on ribosome-sequencing (Ribo-Seq) profiling. In parallel, the recognition of putative negative data, such as the intron-containing transcripts that associate with ribosomes, remains remarkably low, indicating that D-sORF could be efficiently applied to filter out false-positive sORFs from Ribo-Seq data because of the non-productive ribosomal binding or noise inherent in these protocols.

## 1. Introduction

Small open reading frames (sORFs; <100 amino acids) are widespread across all genomes. Their predominantly non-genic location also contributes to the low conservation scores, traditionally used to indicate functional significance in short sequences [1]. Advances in recent decades have revealed various peptides encoded by sORFs in various organisms, from bacteria to humans [2,3,4,5,6,7,8,9,10,11,12,13,14,15,16,17,18,19]. These studies have illuminated the roles of microproteins encoded by sORFs in regulating essential biological processes, including metabolism [7,13], DNA damage response [20], endocytosis [21], immune surveillance [22,23], development [10,14], and cell death [12]. For instance, specific microproteins have been implicated in fine-tuning metabolic pathways and the orchestration of cellular responses to DNA damage [20], highlighting their potential as therapeutic targets and biomarkers.

The classification of long intergenic, interleaving, and antisense non-coding transcripts (lncRNAs) has traditionally used an arbitrary cut-off ORF length of fewer than 100 amino acids. It also relies on the low scores from alignment-based algorithms for nucleotide and protein conservation. As a result, many lncRNAs may have been erroneously misclassified as non-coding [24]. An emerging understanding is that lncRNAs have risen relatively recently in evolution and may represent a de novo source of novel protein-coding genes [25]. These transcripts share all the features of coding genes (Cap, polyA, 5′, and 3′ UTRs). Still, they tend to harbor much smaller ORFs, contain fewer introns, exhibit weak sequence constraints, and show limited phylogenetic conservation [26,27,28]. To date, protein-coding and functional sORFs have been found in various transcripts. These include untranslated regions of mRNA (5′ and 3′ UTRs), lncRNAs, and microRNA transcripts (pri-miRNAs) [19,29,30,31].

Advances in ribosome-sequencing profiling (Ribo-Seq) [32,33,34] and proteomics [35,36] provided the tools necessary to identify protein-coding sORFs. They revealed that the translation of non-coding regions could also be pervasive [37,38]. Various computational methods for ribosome-profiling data have been developed that rely upon specific distribution features of ribosome footprints, like the ORF and FLOSS scores [39,40,41], and allow the genome-wide identification of many long non-coding RNAs (lncRNAs), 5′ and 3′ UTRs [37,38] associated with ribosomes [39,42,43]. Sequencing of ribosome-protected mRNA fragments (RFPs) from samples treated with antibiotics that halt ribosomes at translation initiation sites (TIS) [44] allows the identification of sORFs with experimentally validated TIS positions. However, these datasets have only recently begun to be compiled compared to traditional antibiotics, such as cycloheximide [41]. During the last few years, corresponding public resources have been established, like the GWIPS-viz browser [45], smProt [46], OpenProt [47], and www.sorfs.org (1 March 2023) [39,40,41] databases, to correctly classify sORFs and discover novel micropeptides.

Many lncRNAs, as well as 5′ and 3′ UTRs of mRNAs [37,38], have been reported to show ribosome-profiling patterns similar to canonical non-coding RNAs (e.g., rRNA) that are known not to be translated, implying that many lncRNAs are unlikely to produce functional peptides [48]. However, the ribosome footprints and the translational machinery heavily depend on the transcript abundance and cellular types and states. Genomes undergo widespread transcription during stress to produce a diverse range of non-coding RNAs [49,50,51]. Therefore, a large proportion of sORFs are expressed in response to stress and exported to the cytoplasm to associate with the translational machinery [49,50,51]. This fact may indicate that computational annotation pipelines based on experimental data may inherit a significant range of transcriptional and translational “noise”, which may act as a rate-limiting step toward the identification of unknown small protein-coding genes or other types of regulatory sORFs that could associate with ribosomes to control the translation of other genes [52]. In addition, mass spectroscopy analyses have found many fewer peptides than predicted sORFs, indicating that accurate annotation of sORFs is critical for both their reliable identification and their unbiased nomenclature classification as protein-coding, functional sORFs or biological/technical “noise” [53].

The problem of identifying small coding ORFs was addressed for the first time nearly a decade ago through an algorithm based only on sequence information [54]. Since then, various sORF-specific ab initio prediction pipelines have been implemented. A group used a combination of cross-species conservation analysis and a phylogenetic assessment of the alignment-based mutations for their synonymous or nonsynonymous nature (Ka/Ks values) [18,54,55]. Others used a hexamer composition bias between coding and non-coding sequences at consecutive windows of 30 bp with a step size of 3 bp [56], coupled with a phylogenetic-based assessment of synonymous versus nonsynonymous substitutions rates, to select more functional sORFs [57]. However, alignment-based methods can be limited depending on the corresponding sORF’s length, conservation, and amino acid composition [42], and algorithms comparing coding vs. non-coding sequences have been thus far performed in small windows, not using the sequence features of a translating ORF, like the presence of a translation initiation site (TIS) [44,58].

In this paper, we present D-sORF, a support vector machine-based framework utilizing prediction accuracy supported by a machine-learning model fed with features extracted from the nucleotide composition of the ORF and the sequence information around the start codon, i.e., the translation initiation site (TIS), in a large set of well-established proteins [44]. D-sORF can be applied in a genome-wide manner or just on sORFs associated with ribosomes to classify them based on a large window of sequence identity to the TIS and ORF nucleotide composition without the incorporation of parameters like conservation and phylogenicity. D-sORF calculates and classifies sORFs between 60 and 100 amino acids in length with 97.88% precision and 96.60% accuracy. For sORFs between 33 and 60 amino acids, the precision reaches 94.74%, while the classification accuracy is 92.37%. For sORFs shorter than 33 amino acids, 91.16% precision and 88.15% accuracy are achieved. Remarkably, when D-sORF is applied to a selection of sORFs from the sORFs.org database annotated by both Ribo-Seq data and Ensembl Gene ID (i.e., they represent true microproteins), 935 of the 1075 sORFs are identified as positive, achieving 77.96% accuracy and outperforming all the other computational approaches, including FLOSS.

## 2. Materials and Methods

In this section, we describe the D-sORF framework as well as the datasets used for the evaluation of our method.

### 2.1. Positive Datasets

**Figure 1:**
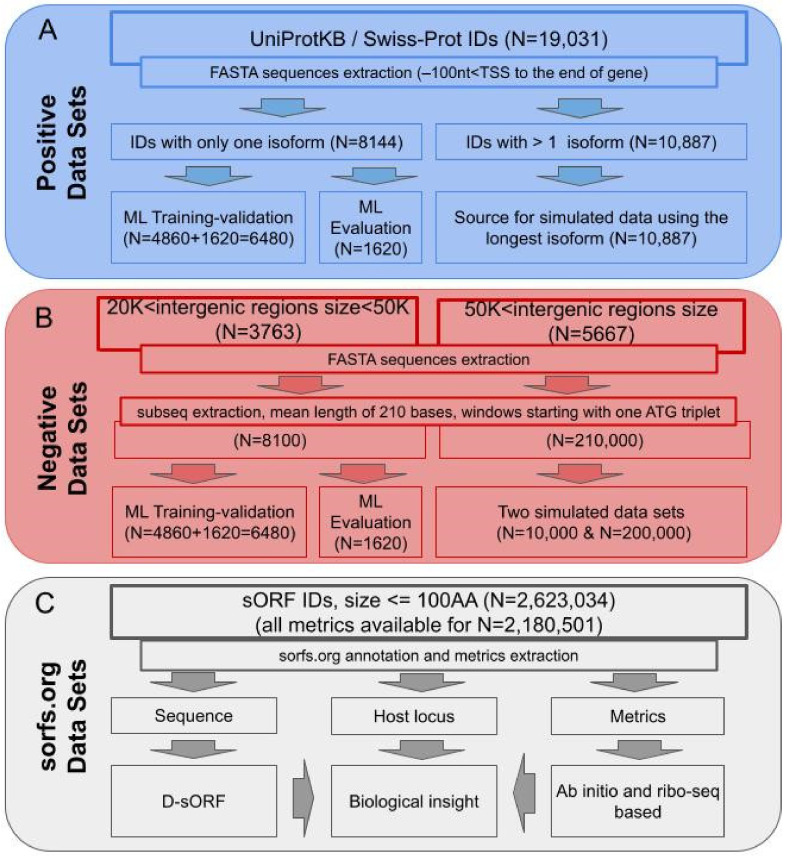
Dataset creation workflow for the datasets used for training and validation of the D-sORF algorithm. (**A**) All the positive datasets originated from protein-coding sequences with an Ensembl ID. For the curated TIS positions, only Swiss-Prot labelled transcripts were selected. The source dataset was split randomly into training and simulation datasets. The training dataset was subsequently parted in ML model training and evaluation subsets. Only one isoform (the longest) per gene was selected for constructing the simulated positive dataset used for validation. (**B**) For the negative dataset construction, intergenic regions, non-overlapping with coding loci, were selected. Two different negative validation datasets were created. One was balanced (N = 10,000) and one imbalanced (N = 200,000) compared to the positive dataset (N = 10,000). (**C**) The sORFs dataset was created from the orfs.org repository by obtaining the corresponding nucleotide sequence for all the sORFs, together with the host region coordinates, annotations and all the metrics (PhyloP, PhastCon, ORFscore, FLOSS) available in the repository. For each sORF sequence, a 100 nts upstream region was also extracted and combined, then forwarded to the D-sORF algorithm. The resulting scores were compared with the algorithmic metrics available in the sorfs.org repository in order to gain biological insight.

The positive datasets exclusively originated from protein-coding regions (Figure 1A). Initially, we identified and retrieved 43,084 protein sequences corresponding to 19,031 unique UniProt IDs from the Ensembl Repository. It is important to mention that we also retrieved sequences with UniProt/Swiss-Prot IDs. Meanwhile, a small upstream region of 100 nucleotides was also retrieved for each sequence to extract features around the translation initiation site.

This initial dataset was split into two parts. The first part consisted of 8144 coding sequences with only one isoform in Swiss-Prot to avoid unnecessary complexity deriving from alternative start sites. This first part was retained for the machine-learning component of the algorithm. It was divided into two non-overlapping sets for training and testing, respectively (see Section 2.6). The second part of the initial dataset (i.e., the remaining 34,940 sequences) was used to construct a positive simulated sORF dataset, which corresponded to 10,887 genes with alternative isoforms, from which the longest isoform for each gene was selected as the source sequence (for simplicity).

### 2.2. Negative Datasets

The negative dataset (Figure 1B) was constructed from the RefSeq database (hg38) [59], where the gene-mediated regions’ coordinates and sizes were annotated. To avoid the inclusion of sequences rich in functional information (i.e., promoters), the first 2000 bases upstream of each gene were discarded. Next, the intergenic space was used to extract the negative datasets for training and evaluation. The negative dataset for training was extracted from the 20 K to 50 K size intergenic regions. To ensure that the negative training dataset was balanced with the positive dataset (as described above), 8100 non-annotated/non-functional regions starting with ATG and a mean length of 210 bases were extracted. Regions larger than 50 K in length were utilized (not overlapping with the training datasets) to extract 210,000 sequences split into two datasets of 10,000 and 200,000 (balanced and unbalanced relative to the positive simulated dataset, respectively) to be used as simulated negative datasets. Since the identification of functional sORFs is known to be a heavily imbalanced task, the second negative simulated sORF dataset was constructed to be over 20 times larger compared to the positive simulated sORF dataset (i.e., 10,000 sequences).

### 2.3. Small ORF Datasets

Data for the Ribo-Seq-derived sORFs (Figure 1C) were obtained from sorf.org, a public repository for sORFs identified by ribosome footprint sequencing. The analysis encompassed human sORFs with lengths of fewer than 100 AAs. Among the various information provided by the repository, the corresponding annotation regarding the sORF’s host genomic region was used to group the sORFs as exonic (located in the coding sequence of a gene), intronic (located in the intronic part of a gene), intergenic (sORFs located between genes), ncRNA (sORFs located on non-coding RNA), 3′-UTR (sORFs located in the 3′-UTR), 5′-UTR (sORFs located in the 5′-UTR), NMD (nonsense-mediated decay; if the coding sequence, following the appropriate reference of a transcript, finishes >50 bp from a downstream splice site, then it is tagged as NMD), TEC (to be experimentally confirmed; used for non-spliced EST clusters that have polyA features), NSD (non-stop decay; transcripts that have polyA features, including a signal, without a prior stop codon in the CDS), and sORFs (sORFs corresponding to Ensembl-annotated protein-coding ORFs of fewer than or equal to 100 AAs). The latter subgroup was a biological positive dataset representing true coding sORFs. From the 2,623,034 downloaded records, sorfs.org provides (FLOSS score [37], ORFscore [39], PhyloP [55], and PhastCon [60]) metrics for 2,180,501 sORFs. We selected the last mentioned for a detailed investigation.

### 2.4. D-sORF Machine-Learning Framework

**Figure 2:**
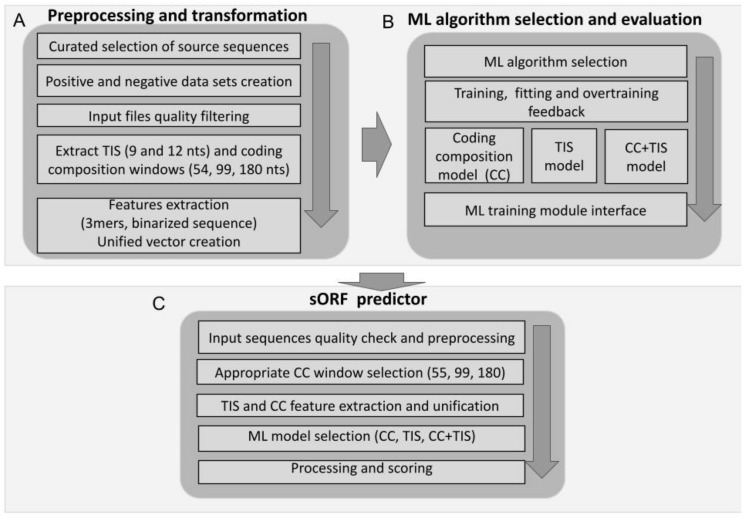
Workflow of the D-sORF Framework. (**A**) In the “preprocessing and transformation” module, the input sequence datasets were cleared from ambiguous and low-quality sequences and sequence windows for the TIS and CC (coding composition) were separated for feature extraction. A vector was created for all the CC and TIS features individually as well as from the unification of the corresponding TIS and CC features. These vectors were the drivers of the training process. (**B**) In the “ML algorithm selection and evaluation” module, comparison and selection between well-known ML algorithms (e.g., SVM, RF) took place. For each feature vector (CC, TIS and combined CC with TIS), a separate output model was trained to be used from the D-sORF main module (predictor). (**C**) The D-sORF predictor was fed with sORFs for processing. The best fitting window among 55, 99, and 180 was selected based on the sORF length. The user could select which model to apply (CC, TIS or combinatory CC+TIS mode) The Diana-sORF returns a score in the range [0 to 1]. Higher scoring values provide the sORF with a greater chance of being coding.

D-sORF is a modular machine-learning framework consisting of three sequential components, namely data preprocessing and transformation, machine-learning algorithm selection and evaluation, and sORF predictor (Figure 2).

### 2.5. Data Preprocessing and Transformation

The data preprocessing and transformation module takes as input the positive and negative datasets in standard FASTA format (Figure 2A). This module analyzes the raw FASTA data, transforming them into the appropriate internal structures while performing the essential quality control to exclude unqualified sequences, e.g., sequences with ambiguities (only A, C, T, and G are allowed). Following this essential initial step, the module continues the feature extraction process, extracting two separate sequence windows for the TIS and coding composition (CC) sampling regions to score two separate features for the coding potential (Supplementary Figure S1). The TIS window includes nucleotides at positions −7 to +5 centered at the given start codon of the sORF sequence. It represents the translation initiation site (TIS) pattern, excluding the start codon and thus avoiding the potential bias of start codon usage in sORFs. Subsequently, each nucleotide window is transformed into a new binarized representation based on the code A = 0001, C = 0010, T = 0100, and G = 1000 (binary sequences with the greatest Hamming distance), resulting in a 36-element vector.

To capture the coding composition (CC) of a given sORF, three different sequence length CC windows of 54 nts, 99 nts, and 180 nts were extracted downstream of the first +90 nts until the corresponding stop codon. The first 90 nts were omitted from training and testing to avoid plausible signal peptides known to precede the protein-specific nucleotide context [61]. Signal peptides have a very specific amino acid composition [2] and can produce a negative bias during the training procedure. All the windows were extracted on a 3-step basis to maintain the in-frame information. Subsequently, a 64-element vector was created containing the frequency of each of the 64 different 3-mers. Finally, the TIS and CC-derived vectors were unified.

### 2.6. ML Algorithm Selection and Evaluation

The initial dataset, consisting of 16,200 examples (8100 positive and 8100 negative), was randomly split to create the training (9720—60%), validation (3240—20%), and test (3240—20%) subsets. The positive–negative ratio was maintained in the datasets produced above. Three well-known machine-learning algorithms were investigated, namely Support Vector Machine (SVM) [62], k-Nearest Neighbor (k-NN) [63], and Random Forest (RF) [64].

To ensure unbiased model selection based on performance, the hyper-parameter space of the algorithms was tested for the best combination toward the best cross-validation score. The hyper-parameters optimized for SVM were (a) the kernel type used in the algorithm, (b) the kernel coefficient gamma, and (c) the penalty parameter C. The hyper-parameters optimized for k-NN were (a) the number of neighbors, (b) the weight function used in the prediction, and (c) the algorithm used to compute the nearest neighbor. Finally, the hyper-parameters tuned for RF were (a) the number of trees in the forest, (b) the function to measure the quality of a split, and (c) the number of features to consider when looking for the best split.

The search in the hyper-parameter space was performed exhaustively, considering all the parameter combinations. The tuning of the hyper-parameters was performed on the training data using 10-fold cross-validation. After obtaining the best hyper-parameter combination for each algorithm, the three algorithms (SVM, k-NN, RF) were re-trained on the training set using the selected hyper-parameter combination. The final accuracy of each model was measured using the initially reserved test set. Trained models compose the D-sORF predictor core. SVM (RBF kernel) achieved the best performance (ACC = 98.1%), followed by RF (ACC = 97.5%), and finally, k-NN (ACC = 94%). Hence, the analysis proceeded using the SVM classifier. To obtain the probabilities for each respective label, the parametric approach based on Platt’s sigmoid model [3] was used, based on fitting a logistic regression model (sigmoid) to a classifier’s scores. The validation set was used for the probability calibration, which differs from the model-fitting dataset.

### 2.7. sORF Predictor

**Figure 3:**
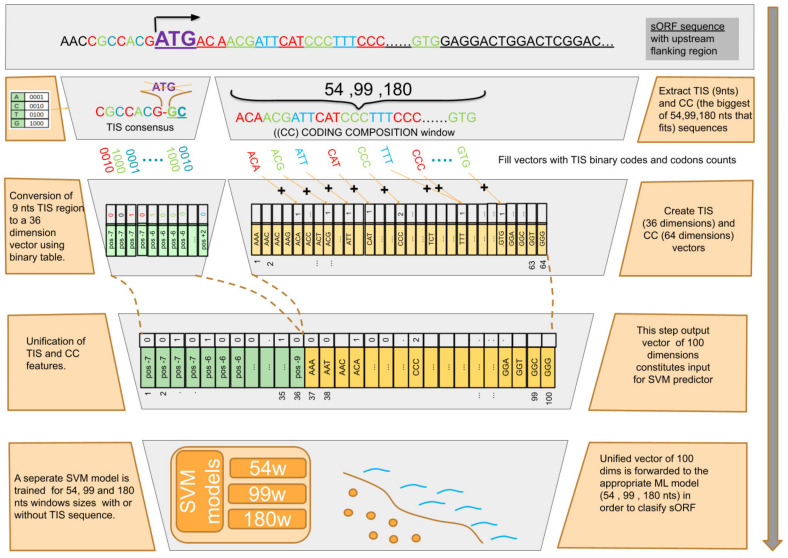
Workflow of the D-sORF predictor. The signal peptide on the sORF sequence is not excluded for the CC sequence (user can enable this feature). The start codon is removed in order to extract the TIS pattern sequence. Both the TIS and CC features are used for the classification and automatic selection of the best fitting window sequence (54, 99, 180) and ML model, according to the sORF length. Processing starts using as an input the sORF sequence combined with the upstream flanking region. After that, the TIS (9 nts) and CC (the biggest among 54, 99, 180 nts) sequences are extracted. The TIS sequences are then converted to a 36-dimension vector using the same binary table as in the training module. This constitutes the first part of the final 100-dimension vector, while the coding composition 64-dimension vector constitutes the second part. The derived vector is then forwarded to the appropriate ML model (54, 99, and 180 nts) for the sORF to be classified.

The final standalone algorithm of the D-sORF framework is the sORF predictor (Figure 2C), which is based on the trained ML model and can be applied for the classification of a given sORF. Similar to the training and evaluation module, two types of windows were selected for the TIS and CC feature extraction (Figure 3). The CC window can be 54, 99, or 180 nts according to the sORF length. In the default operation mode, the algorithm selects the longest windows for feature extraction but allows this option to be parameterized by the user. After that, the TIS and CC features are concatenated inline, resulting in a 100-dimension feature vector, which is classified by the appropriate ML model of 54 nts, 99 nts, or 180 nts, respectively, returning a score ranging from 0 to 1. The user can configure the D-sORF predictor with any trained models, depending on the sORF length range under study.

The downstream analysis proceeds by incorporating big data originating from sorfs.org. It produces a series of reports that categorize the outputs according to the annotation, host region, D-sORF classification, and all the metrics for encoding the ability or classification parameters provided by the repository.

## 3. Results

Two different approaches were employed for the evaluation of D-sORF. Specifically, different types of simulated datasets were used for the initial algorithmic evaluation of D-sORF. Furthermore, the biological significance of D-sORF was evaluated by large-scale analysis of experimentally validated sORFs and by comparing D-sORF’s performance to the available metrics for coding potential (FLOSS score, ORFscore, PhyloP, PhastCon) and to the different sORF annotation classes. The Ensembl ID-labeled class of sORFs annotated as small proteins was used as the most representative biological positive dataset, while the sORFs annotated as introns were selected as a biological negative dataset.

### 3.1. Algorithm Evaluation

To evaluate the predictive strength of the classification models created from the features, the TIS and CC alone or in combination, the algorithm was tested using the following seven sets of features: TIS on its own, CC on its own with three different length windows (54, 99, and 180 nts), and combinations of TIS with each of the different length CC windows. When applying the problem of recognizing coding sORFs on real data (genome, transcripts, and ribosome-associated transcripts), the ratio of positive to negative data is heavily unbalanced, with negative data usually being over-represented. To partly model this imbalance in our evaluation procedure, two different pairs of validation datasets were created: the first pair represented a balanced combination of 10,000 positive with 10,000 negative samples, whereas the second contained an over-represented negative dataset (200,000), i.e., twenty times larger than the positive set.

**Figure 4:**
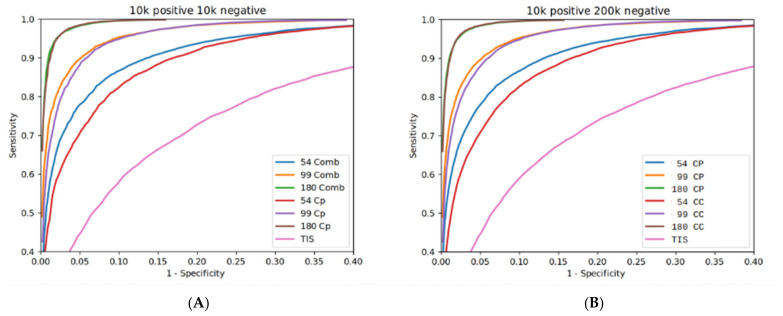
D-sORF ROC curves. Sensitivity vs. specificity diagrams following integration of the TIS and CC features into a unified coding potential (CP) feature. The diagrams depict the framework performance after the integration of the TIS in 54, 99, and 180 nts CC window mode (**A**) for balance data, and (**B**) for imbalanced data.

We performed two sets of evaluation procedures, one for each balanced and one for each unbalanced group of datasets. In each experiment, we compared the seven previously mentioned models (TIS, CC54, CC99, CC180, CC54+TIS, CC99+TIS, CC180+TIS). In all cases, the classifiers with the combined features performed better compared to the CC and TIS alone. For the balanced dataset, the classification of an sORF using only the smallest size, CC54, reached 0.87 precision, 0.86 sensitivity, 0.87 accuracy, and 0.87 specificity (Supplementary Table S1A). When the algorithm encompassed the TIS features (i.e., CC54+TIS), the precision, sensitivity, accuracy, and specificity of D-sORF changed to 0.91, 0.85, 0.88, and 0.92, respectively (Supplementary Table S1B). The other two models, CC99 and CC180, presented similar improvements (Supplementary Table S1C–F). The combined model was termed CP to represent the unified coding potential feature. Since it showed the best performance, we may conclude that both features (TIS and CC) have added value when combined, thus highlighting the potential role of the TIS in sORFs’ encoding ability (Supplementary Figure S2). The trade-off between sensitivity and specificity and the differences between CC and CP were demonstrated using ROC curve diagrams (Figure 4).

Similarly, in the unbalanced dataset, the classifiers exhibited the same behavior as in the balanced set. The unified model CP, combining the two different features (i.e., TIS and CC180), performed better and exhibited the best performance compared to the TIS and CC models alone. CC180+TIS achieves 0.69, 0.95, 0.97, and 0.97 (0.63, 0.96, 0.97, and 0.97 for CC180), while CC99+TIS shows 0.47, 0.90, 0.95, and 0.95 (0.40, 0.91, 0.93, and 0.93 for CC99) for precision, sensitivity, accuracy, and specificity (Supplementary Table S1I,J,K,L). The CC54+TIS model shows 0.34, 0.84, 0.91, and 0.92 for the same metrics (0.26, 0.85, 0.88, and 0.88 for CC54) (Supplementary Table S1G,H). Based on these results, we can conclude that although the larger the CC window, the better the performance, D-sORF retains good scores for sensitivity and specificity for all the window sizes (Supplementary Figure S2).

It is worth noting that D-sORF is a threshold-enabled algorithm, and users can adjust the threshold for fine-tuning. All the above metrics were obtained using a threshold value of 0.5 (scores above 0.5 count as coding). The sensitivity and specificity are balanced for a threshold of 0.9. Using this value significantly increases the algorithm’s precision. For instance, the precision in the unbalanced dataset reaches 0.68 from 0.34 for CC54+TIS, 0.77 from 0.47 for CC99+TIS, and 0.87 from 0.69 for CC180+TIS (Supplementary Table S1H,L).

### 3.2. Application of D-sORF in Annotating Experimentally Detected sORFs in Genomic Regions Enriched with Coding and Non-Coding Transcripts

D-sORF’s performance on real-life sORFs was tested using the sorfs.org repository of experimentally validated sORFs regarding their start codon and association with the ribosome footprinting coding patterns. In addition, the sORFs used were already annotated by sorfs.org regarding their location around or within the main mRNA coding frame, as annotated by Ensembl. Exonic sORFs are located in the exons of mRNAs but out of frame, while intronic sORFs are in the introns of mRNA genes. Moreover, 5′ and 3′ UTRs represent sORFs located in mRNAs’ corresponding exonic but non-genic regions. LncRNAs, TEC, pseudogene, and NMD groups are sORFs located in transcripts annotated by Ensembl as Long Non-Coding RNAs, as To-be-Experimentally Confirmed protein-coding, as Pseudogenes, and as transcripts with premature stop codons likely to be degraded through the Non-sense Mediated Decay pathway, correspondingly. Finally, sORFs detected in lncRNAs but located at distal genomic sites (>5000 bp ± of any mRNA) were studied separately from the rest of the lncRNAs, as Intergenic (Supplementary Figure S3). The above biotype groups contain transcripts harboring a spectrum of expected or known coding/non-coding capacity. Based on current knowledge, the intronic regions show the most biological relevance as a negative control (highly non-coding group).

At the same time, the 5′ UTR sORFs represent an established genomic location of a known functional subclass of sORFs, named upstream ORFs (uORFs), with an established role in mostly repressing but also, for a subset of them, regulating the translation of the mRNA ORFs [65]. However, we considered 5′ UTR sORFs as a test group, not as a biological positive reference group of genomic regions with mainly and highly coding sORFs. Instead, we selected a subset of transcripts from the sorfs.org database corresponding to the Ensembl IDs of validated microproteins (i.e., <100 amino acids).

**Figure 5:**
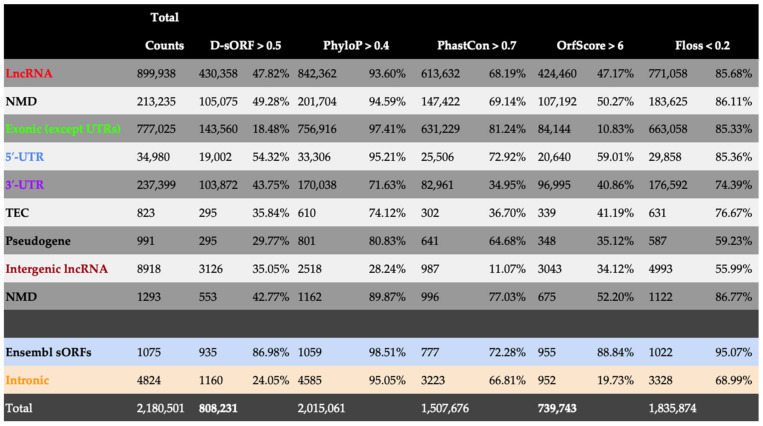
Genome-wide application of D-sORF to classify experimentally validated sORFs as coding. The table contains all the sORFs from the sorfs.org database and shows the absolute count and percentage of sORFs classified as positive by D-sORF, PhyloP, PhastCon, ORFscore, and FLOSS at their corresponding cut-off values. The sORFs have been annotated based on sorfs.org classification parameters as exonic (sORFs located in the exonic part of a gene), intronic (sORFs located partially or completely overlapping introns), intergenic (sORFs in lncRNAs at distal sites > 5000 bp), lncRNA (sORFs overlapping long non-coding RNAs), 3′ UTR (sORFs in 3′-UTR regions of genes), 5′ UTR (sORFs in 5′-UTR regions of genes), Ensembl sORF (sORFs corresponding to Ensembl ID protein-coding ORFs of fewer or equal to 100 AA), NMD (classified as nonsense-mediated decay if sORF overlaps the coding sequence of a transcript that finishes >50 bp from a downstream splice site) and TEC (to be experimentally confirmed as protein-coding, overlapping EST clusters that have polyA features). The colors associated with each sORF class indicate the genomic region from which they originate, as illustrated in Supplementary Figure S3.

The resulting D-sORF scores across all the above annotation groups were compared to all the available classification metrics used in the sorfs.org database to classify the detected sORFs’ coding potential, such as the FLOSS score, ORFscore, PhyloP, and PhastCon (Figure 5). The PhyloP and PhastCon scores are based on phylogenetic information, while the ORFscore and FLOSS scores algorithmically analyze different distribution parameters from ribosome-footprinting experiments. For each type of score, the cut-off values suggested in the corresponding reference papers on sorfs.org were used [40,41]. The PhyloP and PhastCon algorithms showed a relative constitutive detection rate across all the annotation groups, including the positive (Ensembl sORFs) and negative (Intronic) groups. The PhyloP system scored for the coding sORFs with an increased frequency, while PhastCon had a decreased frequency rate in both of the above groups compared to the corresponding detection rates of the experimentally-based algorithms of ORFscore and FLOSS.

In contrast, D-sORF detected coding sORFs across all the groups (including positive and negative groups) with a frequency rate almost similar to the experimentally based algorithm ORFscore. In addition, D-sORF’s classification of coding sORFs showed a comparable detection rate to the experimentally based FLOSS score with some important discrepancies, which could be attributed to the increased sensitivity of the FLOSS score, as indicated by its increased detection rate (68.99%) within the intronic negative group. Regarding the positive group of annotated micropeptides (Ensembl sORFs) contained in the sorfs.org database, D-sORF scored as positive 935 out of 1075 (86.98%), showing similar discriminatory ability to ORFscore (955 out of 1075, 88.84%) and proving its biological significance in identifying true-positive peptide-encoding sORFs. A representative subset is presented in Appendix A.

Reassuringly, D-sORF, unlike PhyloP, PhastCon, and FLOSS, scored low (~24%) in the mainly non-coding class of intronic sORFs, again similarly to the experimentally based ORFscore (~20%), establishing its biological relevance in understanding coding potentiality in rare or newly evolved sORFs. It is important to note that the 5′ UTR sORFs showed the second to highest positivity detection after the Ensembl sORFs by both D-sORF (~54%) and ORFscore (~59%) (Figure 5). In summary, D-sORF performed significantly better (as ORFscore) than the FLOSS score. For example, in the lncRNA class of sORFs, which is expected to be enriched for peptide-encoding sORFs, D-sORF showed a comparable prediction score (47.82%) to ORFscore (47.17%). In comparison, FLOSS and PhyloP produced significantly greater positive counts (85.68% and 93.60%) for lncRNAs.

**Figure 6:**
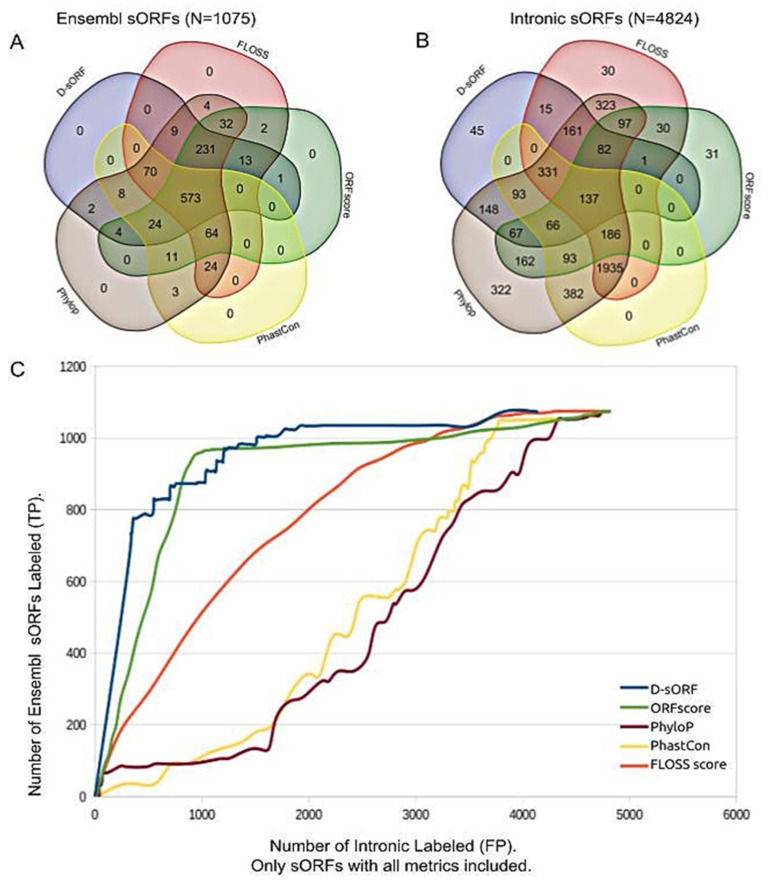
Evaluation of D-sORF compared to all the metrics available in the sorfs.org repository. Venn diagram of (**A**) Ensembl-validated sORFs and (**B**) intronic sORFs. Presenting the results of all the different algorithms and metrics. Counts and percentages of sORFs that were classified as positive and are in common between D-sORF, PhyloP, ORFscore, and FLOSS are shown. Only sORFs with values from all the scores were used. (**C**) The relation of the prediction from the Ensembl-annotated sORFs and intronic sORFs at different thresholds for D-sORF, PhyloP, ORFscore, and FLOSS.

To gain more insight, 1075 Ensembl sORFs and 4848 intronic sORFs with prediction scores by all the metrics were plotted in Venn diagrams. In the Ensembl sORF positive group, significant overlapping of common positive sORFs was observed (573/1075 ~54%) for all the metrics and (804 ~75%) for D-sORF, ORFscore, and FLOSS combined (Figure 6A). On the contrary, in the intronic category, slight overlapping of common positive sORFs (137/4824 ~3%) was observed (Figure 6B). It remains to be investigated whether the above small percentage of intronic sORFs that were detected as positive by all the metrics and by D-sORF, ORFscore, and FLOSS score combined could represent a subset of circular RNA transcripts that translate microproteins with significant biological roles in human diseases [66].

Importantly, using the Ensembl-labeled sORFs as a positive set and intronic sORFs as a negative one, D-sORF’s biological specificity surpassed PhyloP, PhastCon, and FLOSS score both in accuracy (77.96% vs. 22.00%, 40.31%, and 42.69%) and precision (44.63% vs. 18.76%, 19.43%, and 23.49%). The metrics are slightly lower than ORFscore. Statistical measures for all the metrics are presented in Supplementary Table S2.

Finally, all the metric scores for the Ensembl and intronic sORFs were plotted against each other. Especially for PhyloP and ORFscore, we normalized and shifted to the 0–1 interval to have comparable values. Figure 6C shows that D-sORF performed significantly better than FLOSS score (the main computational classification tool used in processing ribosome profiling information) and surpassed all the other algorithms/metrics. The above results indicate that even though the D-sORF algorithm is purely trained on in silico datasets with strictly ab initio computational processes, it can accurately predict coding sORFs better than experimentally based Ribo-Seq algorithms and alignment-based frameworks. In essence, D-sORF can be applied as an unbiased sORF classifier that can be used alone or with other metrics to functionally classify transcripts and describe various biological aspects of sORF classes.

## 4. Discussion

Our knowledge of the translational “vocabulary” of the human genome has expanded thanks to ribosome profiling, or “Ribo-seq”, which has identified hundreds of open reading frames (ORFs) within long non-coding RNAs (lncRNAs) and putative untranslated regions (UTRs) of protein-coding genes. Reference gene annotation programs, however, have been cautious in incorporating these ORFs due to unknowns regarding their physiological roles and experimental repeatability. Nonetheless, it is evident that many “Ribo-seq ORFs” have physiological significance, whereas others control gene regulation or produce stable proteins. In the end, the lack of a standardized ORF annotation pipeline has produced a vicious cycle: studies looking into the roles of Ribo-Seq ORFs will be impeded by their lack of recognition as long as reference annotation databases continue to ignore them [67].

Here, we present D-sORF, which could be used alone or in combination with experimental-based metrics to filter down candidate encoding sORFs from different annotation groups and, therefore, increase the rate of their experimental validation. In addition, D-sORF can be used to discriminate ORFs that associate with ribosome footprint patterns but show true-negative coding potential and thus may have a non-coding regulatory function instead. D-sORF’s significant advantage is that it can predict positive and negative sORFs equally well. Thus it can be applied to functionally classify lncRNAs and other non-coding transcripts that either encode sORFs and thus represent a recently evolved class of protein-coding genes or regulate the translational machinery in a non-coding manner.

D-sORF is an ab initio scoring system that accurately distinguishes coding from non-coding sORFs based on the nucleotide context of a 180 nts coding ORF composition window and a 9 nts window around the TIS of experimentally validated protein-coding ORFs, compared to windows of similar length in carefully curated intergenic regions used as negative datasets. D-sORF represents the first-of-its-kind classification algorithm powered by trained machine-learning models to predict putative translating sORFs in the human genome accurately. Compared to phylogenetic and sequence conservation approaches, like PhyloP and PhastCon, D-sORF exhibited superior performance in accurately depicting, through prediction, the sORF encoding resemblance to protein ORFs. This indicates that, in general, ab initio nucleotide similarity approaches are more effective than cross-species comparison pipelines in utilizing the sequence information of recently evolved small ORFs with little similarity to known proteins and low conservation in the amino acid sequences.

Specifically, PhastCon and PhyloP showed a general trend to predict low or high scores for all the sORFs regardless of their annotation class, including the negative group. For instance, although PhyloP scores high for Ensembl sORFs, it also scores high for intronic sORFs (~94%), showing ~5% specificity and 22% accuracy (see Supplementary Table S2). However, D-sORF, FLOSS, and ORFscore performed with the highest accuracy rate and represented the top three algorithms with the highest prediction accuracy, which were able to discriminate true positives from the Ensembl sORF class. Significantly, these three algorithms also scored the lowest for the best-known negative set of the intronic sORF class.

Furthermore, although D-sORF is based only on sequence training, it showed better discriminatory ability than the Fragment Length Organization Similarity Score (FLOSS), representing the most recent Ribo-Seq algorithmic scoring described by [37]. This algorithm identifies true ribosome footprints by measuring the magnitude of disagreement between the RPF-length distribution of Ensembl-annotated protein-coding sequences and the RPF-length distribution of an sORF. D-sORF’s performance is similar to ORFscore, another Ribo-Seq sORF annotation algorithm that, in contrast to the FLOSS system, is known to have stringent parameters for detecting true ribosome footprints outside the annotated CDS of protein-coding genes (i.e., in lncRNAs, 5′ and 3′ UTR regions) [39]. This indicates that Ribo-Seq-based algorithms might suffer from a translational bias, depending on the pattern of translational efficiency they score.

Our results show that D-sORF showed remarkable prediction sensitivity and was comparable only to Ribo-Seq-based algorithms. Nevertheless, D-sORF’s ab initio approach and carefully curated positive and negative training datasets, coupled with deep-learning models, may represent what is needed to facilitate an alignment-free and data-independent functional classification of experimentally validated sORFs.

D-sORF has significant potential in real-world biological research, particularly in studying low-conservation and/or low-similarity sORFs. These small but crucial genomic elements can encode functional microproteins in various regulatory processes. By accurately predicting coding sORFs, D-sORF can help toward achieving robust annotation, identification, and experimental validation of novel microproteins. This capability, which does not rely on experimental data, is especially relevant in shedding light on previously unexplored aspects of gene regulation and cellular function and understanding diseases where microproteins might play a role in the metabolism, DNA repair, and immune response pathways.

## 5. Conclusions

Our study introduces D-sORF, a machine-learning framework designed to predict coding small open reading frames (sORFs) using the nucleotide context and motif information around the start codons. D-sORF demonstrates high precision (94.74%) and accuracy (92.37%) in identifying sORFs, surpassing traditional and experimental methods such as ribosome sequencing (Ribo-Seq).

D-sORF enhances the prediction accuracy by outperforming conservation-based approaches, making it particularly effective in recognizing recently evolved sORFs with low sequence similarity. Additionally, the tool effectively filters out false positives, such as intronic sORFs, often mistakenly identified by other methods. Its broad applicability allows D-sORF to be applied to various genomic datasets. It provides a robust method for distinguishing coding sORFs from non-coding sequences without relying on parameters like the sequence conservation. Furthermore, D-sORF can functionally characterize non-coding transcripts, potentially identifying novel protein-coding genes or regulatory elements.

Overall, D-sORF represents a significant advancement in genomic research, offering a reliable method for sORF detection that can facilitate discoveries in gene regulation and protein function. This tool holds promise for enhancing our understanding of the genomic landscape and its implications for biology and medicine.

## Data Availability

Dataset available on request from the authors. (https://github.com/nperdi/D-sORF; Contact: arhatzi@uth.gr).

## Supplementary Materials

The following supporting information can be downloaded at https://www.mdpi.com/article/10.3390/biology13080563/s1, Figure S1: Sequence feature extraction windows; Figure S2: Balanced/unbalanced dataset performance; Figure S3: sORF annotation; Table S1: Model performance; Table S2: Algorithm comparison metrics.

## Appendix A

The dataset comprises fifty predicted small open reading frames (sORFs) identified by the D-sORF tool as potentially functional. Each entry in the table includes the unique sORF identifier assigned by sORFs.org, the length of the sORF in nucleotides, and the translated amino acid sequence. The dataset includes sORFs from various studies, providing a comprehensive resource for understanding the functional potential of these small but significant genomic elements. Analysis of the sequences can reveal common motifs, structural properties, and potential roles in cellular processes. All the sORFs in the list are labeled by Ensembl as sORFs, as declared by sORFs.org.

**sORF ID**	**Length**	**Sequence**
SorfId=andreev_2015:538832	300	MVLRRLLAALLHSPQLVERLSESRPIRRAAQLTAFALLQAQLRGQDAARRLQDLAAGPVGSLCRRAERFRDAFTQELRRGLRGRSGPPPGSQRGPGANI
SorfId=elkon_2015:50819	210	MLRWLRDFVLPTAACQDAEQPTRYETLFQALDRNGDGVVDIGELQEGLRNLGIPLGQDAEEVGRRRGAA
SorfId=fritsch_2012:225652	180	MRQLKGKPKKETSKDKKERKQAMQEARQQITTVVLPTLAVVVLLIVVFVYVATRPTITE
SorfId=andreev_2015:496184	213	MAPWSREAVLSLYRALLRQGRQLRYTDRDFYFASIRREFRKNQKLEDAEARERQLEKGLVFLNGKLGRII
SorfId=rubio_2014:98637	141	MKESSIDDLMKSLDKNSDQEIDFKEYSVFLTMLCMAYNDFFLEDNK
SorfId=andreev_2015:629427	261	MTDRYTIHSQLEHLQSKYIGTGHADTTKWEWLVNQHRDSYCSYMGHFDLLNYFAIAENESKARVRFNLMEKMLQPCGPPADKPEEN
SorfId=crappe_2014:52683	249	MSATWTLSPEPLPPSTGPPVGAGLDAEQRTVFAFVLCLLVVLVLLMVRCVRILLDPYSRMPASSWTDHKEALERGQFDYALV
SorfId=calviello_2016:870882	252	MEALGSGHYVGGSIRSMAAAALSGLAVRLSRPQGTRGSYGAFCKTLTRTLLTFFDLAWRLRKNFFYFYILASVILNVHLQVYI
SorfId=andreev_2015:491102	189	MAAATLTSKLYSLLFRRTSTFALTIIVGVMFFERAFDQGADAIYDHINEGVRACAIPDLGPA
SorfId=calviello_2016:375783	189	MTKAGSKGGNLRDKLDGNELDLSLSDLNEVPVKELVSIVPRGPGAHLRRALAPTWATYFPLL
SorfId=liu_HEK_2013:259226	204	MLSRLQELRKEEETLLRLKAALHDQLNRLKVTPRPSPAPPPFLPSDHFPPTWASVVTTCCGQFSATH
SorfId=gonzalez_2014:380995	267	MSIMDHSPTTGVVTVIVILIAIAALGALILGCWCYLRLQRISQSEDEESIVGDGETKEPFLLVQYSAKGPCVERKAKLMTPNGPEVHG
SorfId=calviello_2016:540138	264	MYCLQWLLPVLLIPKPLNPALWFSHSMFMGFYLLSFLLERKPCTICALVFLAALFLICYSCWGNCFLYHCSDSPLPESAHDPGVVGT
SorfId=calviello_2016:185382	207	MAPAAAPSSLAVRASSPAATPTSYGVFCKGLSRTLLAFFELAWQLRMNFPYFYVAGSVILNIRLQVHI
SorfId=andreev_2015:177588	294	MEEISLANLDTNKLEAIAQEIYVDLIEDSCLGFCFEVHRAVKCGYFYLEFAETGSVKDFGIQPVEDKGACRLPLCSLPGEPGNGPDQQLQRSPPEFQ
SorfId=andreev_2015:409703	114	MITDVQLAIFANMLGVSLFLLVVLYHYVAVNNPKKQE
SorfId=andreev_2015:37830	279	MTKLAQWLWGLAILGSTWVALTTGALGLELPLSCQEVLWPLPAYLLVSAGCYALGTVGYRVATFHDCEDAARELQSQIQEARADLARRGLRF
SorfId=andreev_2015:13871	279	MWPVFWTVVRTYAPYVTFPVAFVVGAVGYHLEWFIRGKDPQPVEEEKSISERREDRKLDELLGKDHTQVVSLKDKLEFAPKAVLNRNRPEKN
SorfId=park_2016:1581934	237	MREENKGMPSGGGSDEGLASAAARGLVEKVRQLLEAGADPNGVNRFGRRAIQVAGAPGPRRQGARERGARPRRIGAGT
SorfId=gonzalez_2014:153430	150	VRDKKLLNDLNGAVEDAKTARLFNITSSALAASCIILVFIFLRYPLTDY
SorfId=fritsch_2012:974998	183	MDAVSQVPMEVVLPKHILDIWVIVLIILATIVIMTSLLLCPATAVIIYRMRTHPILSGAV
SorfId=andreev_2015:137699	264	MSTSVPQGHTWTQRVKKDDEEEDPLDQLISRSGCAASHFAVQECMAQHQDWRQCQPQVQAFKDCMSEQQARRQEELQRRQEQAGAHH
SorfId=andreev_2015:321927	207	VGVGKNKCLYALEEGIVRYTKEVYVPHPRNTEAVDLITRLPKGAVLYKTFVHVVPAKPEGTFKLVAML
SorfId=calviello_2016:206180	141	MPAGVPMSTYLKMFAASLLAMCAGAEVVHRYYRPDLVSAGGLLFYF
SorfId=gonzalez_2014:52803	162	VNIDHFTKDITMKNLVEPSLSSFDMAQKRIHALMEKDSLPRFVRSEFYQELIK
SorfId=elkon_2015:525198	114	MVVDRLFLWTFIIFTSVGTLVIFLDATYHLPPPDPFP
SorfId=andreev_2015:272691	150	MDINRRRYPAHLARSSSRKYTELPHGAISEDQAVGPADIPCDSTGQTST
SorfId=calviello_2016:187121	222	LLYLGRDYPKGADYFKKRLKNIFLKNKDVKNPEKIKELIAQGEFVMKELEALYFLRKYRAMKQRYYSDTNKTN
SorfId=loayza_puch_2016:211324	231	MDLRRVKEYFSWLYYQYQIISCCAVLEPWERSMFNTILLTIIAMVVYTAYVFIPIHIRLAWEFFSKICGYHSTISN
SorfId=gonzalez_2014:438290	78	MLDIFILMFFAIIGLVILSYIIYLL
SorfId=cenik_2015:853557	171	MADVSERTLQLSVLVAFASGVLLGWQANRLRRRYLDWRKRRLQDKLAATQKKLDLA
SorfId=andreev_2015:169859	174	MPTGKQLADIGYKTFSTSMMLLTVYGGYLCSVRVYHYFQWRRAQRQAAEEQKTSGIM
SorfId=crappe_2014:5055	144	DVGSLDEKMKSLDVNQDSELKFNEYWRLIGELAKEIRKKKDLKIRKK
SorfId=calviello_2016:673102	225	MFDIKAWAEYVVEWAAKDPYGFLTTVILALTPLFLASAVLSWKLAKMIEAREKEQKKKQKRQENIAKAKRLKKD
SorfId=ingolia_2012:368307	69	LGMEEEDVIEVYQEQTGGHSTV
SorfId=andreev_2015:611100	273	MPKRKAKGDAKGDKAKVKDEPQRRSARLSAKPAPPKPEPRPKKASAKKGEKLPKGRKGKADAGKDGNNPAKNRDASTLQSQKAEGTGDAK
SorfId=elkon_2015:4281	108	MPQTFSGGRGFELDSGSDDMDPGRPRPPRDPDELH
SorfId=calviello_2016:40163	273	MRDPVSSQYSSFLFWRMPIPELDLSELEGLGLSDTATYKVKDSSVGKMIGQATAADQEKNPEGDGLLEYSTFNFWRAPIASIHSFELDLL
SorfId=elkon_2015:564093	213	MPARRLLLLLTLLLPGLGVSDRGAWGGGQLATAGSGPGQRRGAGAGVRAGSATAAARCPVSPAVGGSGRA
SorfId=andreev_2015:217825	87	VERIKERVEEKEGIPPQQQRLIYSGKQM
SorfId=calviello_2016:764879	126	VSKCSEEIKNYIEERSGEDPLVKGIPEDKNPFKEKGSCVIS
SorfId=calviello_2016:642790	204	VKAAFTRAYNKEAHLTPYSLQAIKASRHSTSPSLDSEYNEELNEDDSQSDEKDQDAIETDAMIKVVF
SorfId=andreev_2015:308992	210	MTTLIPILSTFLFEDFSKASGFKGQRPETLHERLTLVSVYAPYLLIPFILLIFMLRSPYYKYEEKRKKK
SorfId=gonzalez_2014:172668	129	VSKAAADLMTYCDAHACEDPLITPVPTSENPFREKKFFCALL
SorfId=andreev_2015:216730	162	VIPKRTNRPGISTTDRGFPRARYRARTTNYNSSRSRFYSGFNSRPRGRVYRSG
SorfId=gonzalez_2014:645009	93	VFRYSLQKLAYTVSRTGRQVLGERRQRAPN
SorfId=werner_2015:456667	147	MVITSENDEDRGGQEKESKEESVLAMLGIIGTILNLIVIIFVYIYTTL
SorfId=iwasaki_2016:288070	114	MAEGNTLISVDYEIFGKVQGVFFRKHTQVCGLQALGW
SorfId=andreev_2015:619791	195	MMNNGLLQQPSALMLLPCRPVLTSVALNANFVSWKSRTKYTITPVKMRKSGGRDHTGGNKDRGI
SorfId=werner_2015:555128	159	MGCHSSKSTTVAAESQKLEEEREGREPGLETGTQAADCKDAPLKDGTPEPKS
