# Supplementary material for: D-sORF: Accurate Ab Initio Classification of Experimentally Detected Small Open Reading Frames (sORFs) Associated with Translational Machinery
Source: Biology (Basel). 2024 Jul 26;13(8):563. doi: 10.3390/biology13080563 (PMC11351124; doi:10.3390/biology13080563)
Supplement: Supplementary file 1 [file biology-13-00563-s001.zip › D-sORF_2024_revision_supplementrary_Figures.pdf]

Supplementary Figures

Supplementary Figure S1

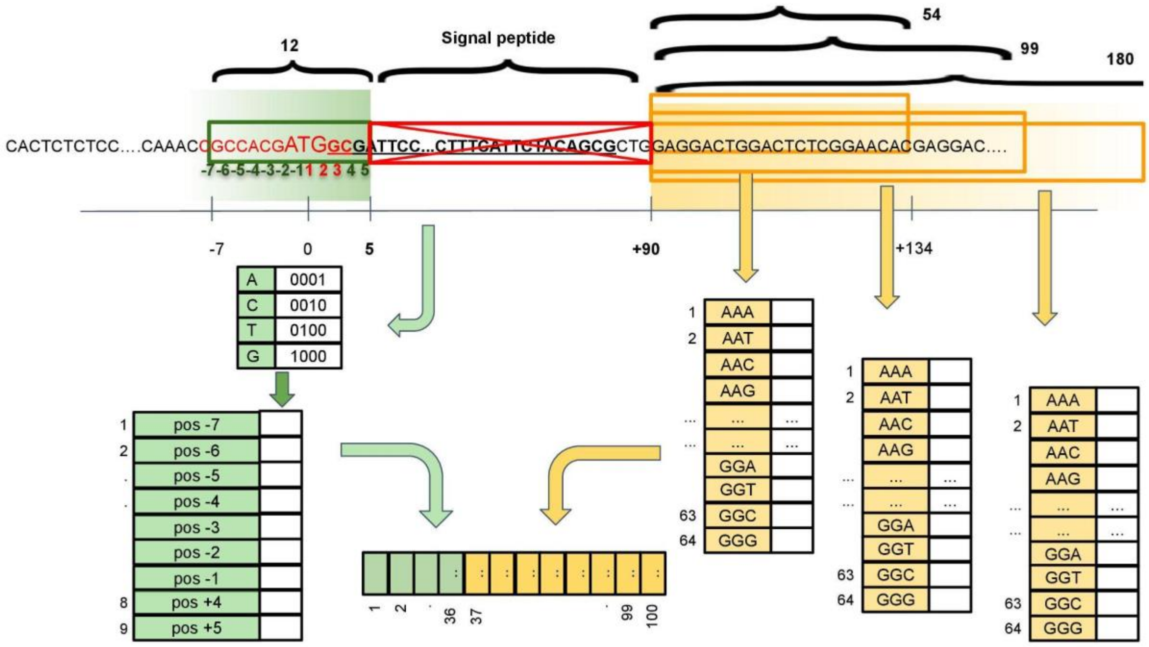

Supplementary Figure S2

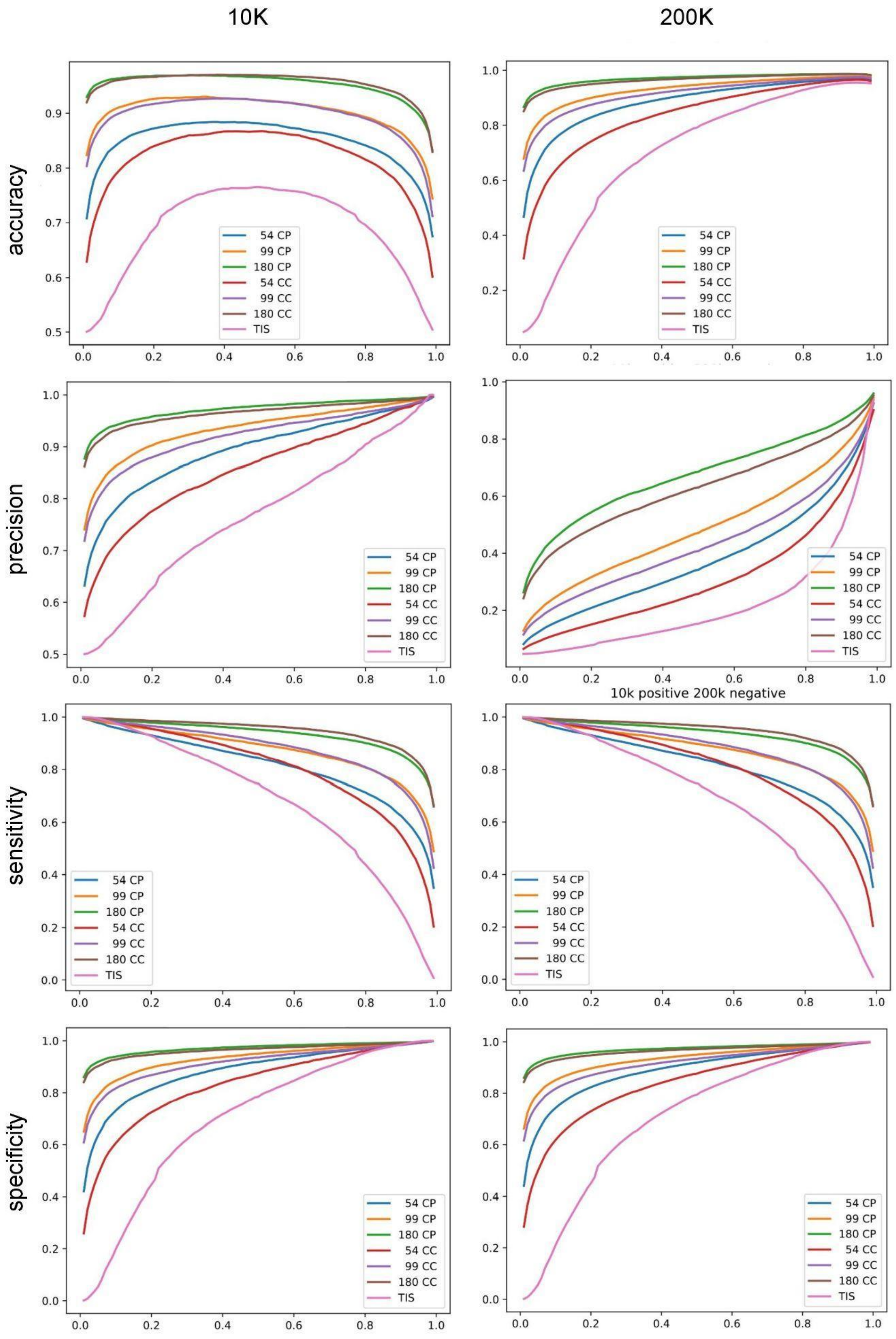

Supplementary Figure S3

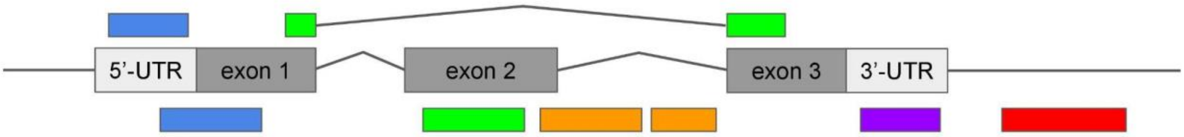

Color	sORF location	Description
(blue)	5'-UTR	5'-UTR sORF, located in the 5' UTR region without exonic overlap
(green)	Exonic	Exonic sORF, located completely in an exonic region
(orange)	Intronic	Intronic sORF, located in an intronic region with or without exonic overlap
(purple)	3'-UTR	3'-UTR sORF, located in the 3;-UTR region
(red)	lncRNAs	lncRNAs, located in-between genes (intergenic)
